# Decarbonization of Power and Industrial Sectors: The Role of Membrane Processes

**DOI:** 10.3390/membranes13020130

**Published:** 2023-01-19

**Authors:** Azizbek Kamolov, Zafar Turakulov, Sarvar Rejabov, Guillermo Díaz-Sainz, Lucia Gómez-Coma, Adham Norkobilov, Marcos Fallanza, Angel Irabien

**Affiliations:** 1 Department of IT, Automation, and Control, Tashkent Chemical-Technological Institute, Tashkent 100011, Uzbekistan; 2 Department of Chemical and Biomolecular Engineering, University of Cantabria, 39005 Santander, Spain; 3 Department of Engineering Technologies, Shahrisabz Branch of Tashkent Chemical-Technological Institute, Shahrisabz 181306, Uzbekistan

**Keywords:** CO_2_ capture, post-combustion, membrane separation, TRL, decarbonization, climate change, CO_2_ capture comparison

## Abstract

Carbon dioxide (CO_2_) is the single largest contributor to climate change due to its increased emissions since global industrialization began. Carbon Capture, Storage, and Utilization (CCSU) is regarded as a promising strategy to mitigate climate change, reducing the atmospheric concentration of CO_2_ from power and industrial activities. Post-combustion carbon capture (PCC) is necessary to implement CCSU into existing facilities without changing the combustion block. In this study, the recent research on various PCC technologies is discussed, along with the membrane technology for PCC, emphasizing the different types of membranes and their gas separation performances. Additionally, an overall comparison of membrane separation technology with respect to other PCC methods is implemented based on six different key parameters—CO_2_ purity and recovery, technological maturity, scalability, environmental concerns, and capital and operational expenditures. In general, membrane separation is found to be the most competitive technique in conventional absorption as long as the highly-performed membrane materials and the technology itself reach the full commercialization stage. Recent updates on the main characteristics of different flue gas streams and the Technology Readiness Levels (TRL) of each PCC technology are also provided with a brief discussion of their latest progresses.

## 1. Introduction

### 1.1. The Problem with CO_2_ Emissions

**Figure 1:**
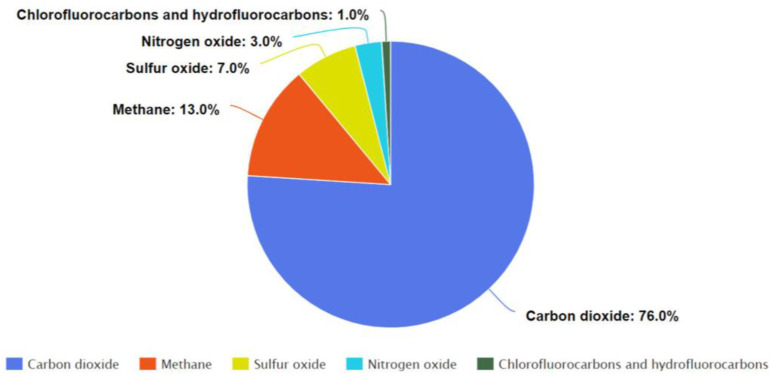
Greenhouse gases emission and their main sources in 2019 (based on the information from [2]).

In the 21st century, there is no doubt that the world is facing the environmental challenge of climate change that has already started its impact on the ecosystem. Greenhouse gas emissions play a crucial role in all global environmental issues. Carbon dioxide is one of the major causes of climate change and corresponds to approximately three-quarters of total greenhouse gases in the atmosphere, as shown in Figure 1 [1,2]. 

CO_2_ emissions mainly come from both natural and anthropogenic sources. Volcanic eruptions, forest fires, the breathing of living organisms, decomposition of organic matter, and ocean release are the most common examples of natural causes, while human activities such as the combustion of biological materials, fossil fuels (primarily coal, natural gas, and oil), and deforestation are all responsible for the rest of the CO_2_ emissions. Although human-caused CO_2_ emissions are significantly smaller than natural emissions, they have disrupted the natural balance maintained for thousands of years prior to human intervention [3]. In order to keep the balance at a reasonable level, the human factor of climate change needs to be minimized.

The Paris agreement, ratified by 196 parties in response to the climate conference held in Paris in 2015, came into force with the aim of maintaining the global temperature increase under 2 °C since the period of pre-industrialization [4]. According to NASA Global Climate Change, in recent decades, the world is experiencing its highest level of CO_2_ emissions, with an increase of about 50% related to human activities since the beginning of the industrial revolution [5]. Despite the unexpected drop in CO_2_’s share in the atmosphere in 2020 by 5.4%, possibly due to COVID-19, in 2021, the global concentration of carbon dioxide in the atmosphere almost returned to a slightly lower level than its record in 2019 and continues to rise [6]. Currently, the atmospheric concentration of CO_2_ has reached over 419 parts per million (ppm), compared to the historical 800,000-year highest CO_2_ level of approximately 300 ppm in 1950 [5]. It is even estimated to increase above 1300 ppm by 2100. This increase in CO_2_ concentration is expected to be associated with a temperature rise of 4 °C if no actions are taken to reduce emissions [7]. 

In early November 2021, during the 26th annual Climate Change Conference held in Glasgow, UK, the participating 197 countries agreed to a new deal of the Glasgow Climate Pact. This Pact reset the goal of the Paris agreement which is to keep the increase of global temperature under 2 °C above the pre-industrial levels and urged to limit the temperature growth to even 1.5 °C so as to take rapid actions in the depletion of global greenhouse gas emissions by 2030. The final decision of the conference was the first to mention fossil fuels as a driver of climate change [8].

**Figure 2:**
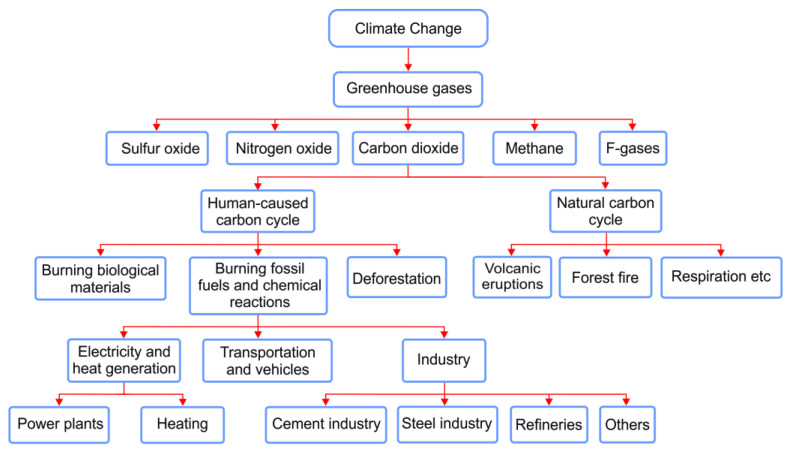
Overview of the main roots of human-related CO_2_ emissions.

Fossil fuels as a driver of climate change are mainly used in the energy and industrial sectors that are responsible for more than half of the global CO_2_ emissions [9]. Over the last few decades, considerable amounts of CO_2_ have been released into the atmosphere as a result of electricity generation, cement and steel manufacture, oil and gas refineries, and the use of fossil fuels in many households [2]. These are illustrated in Figure 2, which provides an overview of the main roots of human-related CO_2_ emissions, and solutions for CO_2_ emission reductions are proposed in the upcoming paragraph.

### 1.2. Solutions for CO_2_ Emission Reductions

**Figure 3:**
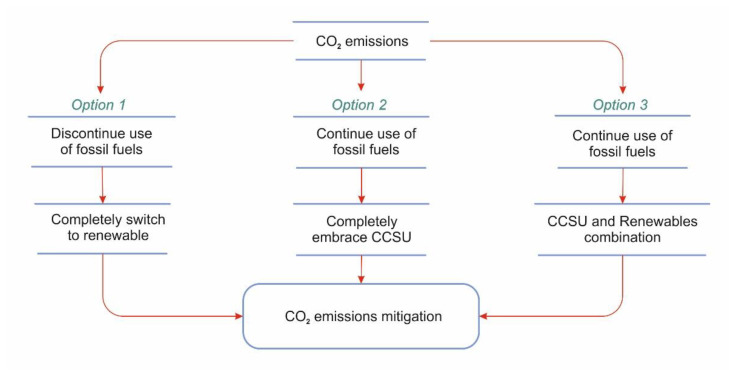
A general roadmap towards minimizing global CO_2_ emissions.

In general, while renewables maintain domination in decreasing emissions, it is uncertain if their falling prices will render other options such as CCSU [10]. Reaching zero carbon emissions by 2050 requires rapid actions in both CO_2_ emission reductions from the large emitting sources by applying CCSU and switching to sustainable energy systems step by step. Therefore, there are mainly three options to mitigate climate change by minimizing CO_2_ emissions, as shown in Figure 3.

According to the systematic analysis above in Figure 2, climate change by human activities is highly dependent on the energy produced by burning fossil fuels. The world’s energy demand and industrial activities increase in relation to global population growth [11]. Therefore, for a sustainable and healthy society, a reliable, environmentally friendly, and efficient energy supply, such as solar, wind, geothermal, hydro, and biomass energy with a green industry is required now more than ever before [12]. We are, however, on the pathway to making the switch from fossil fuel to sustainable energy, which may take a number of decades because of several challenging factors, including the imbalance between energy demand and the existence of renewable energy resources, price and supply fluctuations, technical limitations, and innovative technologies [13]. Thus, a combination of CCSU during the transition to sustainable energy sources cannot be neglected or underestimated.

CCSU is assessed as a promising strategy to mitigate climate change by decreasing the atmospheric concentration of CO_2_ from both power and industrial points [14,15,16,17]. The major human-related sources of CO_2_ emissions are from the industry, transportation, and power generation sectors. Unfortunately, extracting CO_2_ from a conventional transportation source is unfeasible with existing technology, while research is ongoing in this area. Large point sources, such as fossil fuel power plants, the cement and steel industry, chemical plants, etc., should be the main target of capture with existing technology [18]. 

Generally, pre-combustion, oxy-fuel combustion, and post-combustion capture are three primary approaches to the separation of CO_2_ from flue gas [19,20].

Post-combustion CO_2_ capture (PCC) is the most mature and already commercialized approach at a large scale [21]. In post-combustion capture, carbon dioxide is formed mainly with water vapor, nitrogen oxide, sulfur dioxide, and CO_2_ in different ratios by burning fossil fuel. Then, CO_2_ is captured from the flue gas before releasing it into the atmosphere [20]. There has been increased recognition of the post-combustion capture approach in the scientific field due to its simplicity to deploy to existing plants, as there is no need for a significant change to the combustion block [19]. 

PCC systems should be designed according to the emission source specifications, while maximizing process efficiency and minimizing emission reduction costs. Given the wide range of plant sizes and exhaust gas specifications applicable to different emission sources, it is unlikely that a single CO_2_ capture technology will be the best solution for all cases. Therefore, it is convenient to consider several technologies to effectively design the process and select the most efficient and cost-effective option to serve the purpose. It should be also considered that the carbon capture unit normally requires the pre-treatment of the flue gas stream, and therefore it is quite often installed after purification systems such as denitrogenation, desulfurization, and removing dust from the exhaust gas [22]. 

**Figure 4:**
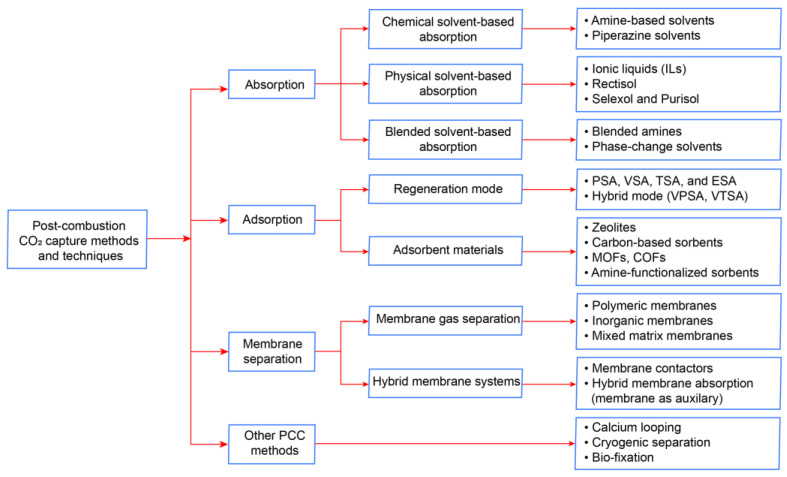
Process technologies for post-combustion capture.

Post-combustion carbon capture can also be classified as solvent-based absorption, cryogenic separation, bio-fixation, membrane separation, solid adsorption, and calcium looping based on their capturing principles (see Figure 4).

Absorption-based carbon capture relies on the principle that CO_2_ in the feed gas is transferred into the liquid phase by selective absorption in a solvent. Solvent-based carbon capture is the most commercially available technology. For instance, amine-based absorption has many advantages such as capturing CO_2_ from low CO_2_ partial pressure flue gases, above 98% CO_2_ product purity, and solvent recovery rates up to 95%. As for adsorption, this process is a chemical or physical process in which molecules, atoms, or ions are captured by a solid adsorbent. Adsorption includes attracting CO_2_ molecules of flue gas on the adsorbent surface. In terms of cryogenic CO_2_ capture, it is a physical separation of CO_2_ based on the differences between the boiling and the desublimation points of CO_2_ in the gas mixture. In this process, CO_2_ is liquefied by condensation at low temperatures and separated from the flue gas. Cryogenic separation technology can obtain higher CO_2_ purity and recovery (99.99%) than other technologies. A calcium looping (CaL) system is a solid looping-based carbon capture system that can be easily retrofitted into the power and industrial sectors. This system uses calciner/carbonator reactors to remove CO_2_ from flue gases using solid calcium oxide (CaO)-based sorbent and regenerate the sorbent back [23]. The carbon bio-fixation method provides natural CO_2_ incorporation into biomass at a relatively low cost in terms of energy. Photoautotrophy and chemolithotrophy are natural mechanisms that have resulted in the consumption of CO_2_ biologically [24]. Algae-based CO_2_ utilization, among others, can be a promising route that uses photosynthesis to capture CO_2_ from flue gas for carbon fixation.

### 1.3. Membrane Technology as Promising CO_2_ Capture Method

New carbon utilization techniques, novel liquid solvents and adsorbents, and membrane materials are being recognized as new CCSU methods. Among them, membrane technology is the fastest developing, in terms of its promising performance. Membrane processes are now one of the most important technologies for industrial separations and are anticipated to play a crucial part in the development of sustainable production systems in the future. Membrane separation belongs to the generation of advanced separation processes following thermal processes, such as distillation and evaporation, and phase processes, such as absorption, adsorption, and extraction [25]. Membrane-based separation is advantageous compared to other methods, with its relatively low environmental impact and the simplicity of scaling up. Apart from that, it can be operated in a continuous system without a need for any solvent/sorbent and its regeneration is preferred by industry over conventional technologies. For these reasons, membranes have been implemented to not only gas separation processes but also several different sectors, including waste-water treatment, natural gas processing, membrane crystallization, pharmaceutical and chemical processes, biogas purification, CO_2_ capture, and other separation processes. The following sections provide more details about membrane separation in PCC, membrane classification, technological maturity of membranes, current challenges, and research advances.

### 1.4. Advances and Novelty of This Review with Respect to the Current State of the Art

This paper provides a detailed systematic analysis of the problems with CO_2_ emissions and pathways to CO_2_ emissions reduction. This is followed by a brief overview of the current state of membrane-based CO_2_ capture technologies, focusing on the bibliometric analysis and different CO_2_ separation techniques. In addition, this review covers updates for membrane-based carbon capture and other major PCC technologies from a broad viewpoint highlighting their main advantages and drawbacks. Apart from that, different types of membrane materials and their gas separation performances, role of membranes, pros and cons of using membranes in PCC, and existing problems based on the real application of membrane technologies are discussed. Updated TRLs of these PCC technologies are provided and discussed with the latest advances and progresses in the field. Finally, various PCC technologies are compared with membrane separation technology based on technological maturity, scalability, economic, and environmental aspects. The technological and economic suitability of each method to the current CO_2_ emitting point sources is also estimated. 

## 2. Search Criteria and Bibliometric Analysis

Bibliometric analysis is a statistical method that quantifies all forms of information carriers and is used to sort out the relationships between search data, as well as clarify the most important ones [26,27]. It is a widely used method for determining a field’s development [28]. Bibliometric analysis and data mining are conducted to study the state of scientific research in the field of membrane-based CO_2_ capture in recent years. For analysis, scientific documents, such as research articles, book chapters, review and conference papers, notes, and business articles, were collected by searching the Scopus web search engine under the terms “membrane CO_2_ Capture”. 

Scientific documents published in the last 10 years were analyzed by document type, number of citations, and keywords. Initially, the scientific works from 2013–2017 and 2018–2022 are compared. Comparative analysis shows that scientific research in this field is developing rapidly. For example, in the period of 3 years between 2020 and 2022, the number of published scientific papers is more than the number released in the 5-year period from 2013 to 2017. This can be explained by the fact that the latest global climate change actions, particularly the Paris agreement in 2015, led to significant attention for environmental protection to become a trend in the scientific network. Between 2013 and 2017, 974 scientific documents were published, followed by 1422 in subsequent years. In order to identify the latest research in the field of membrane-based CO_2_ capture, we refer to the documents published in the years 2017 to 2022.

**Figure 5:**
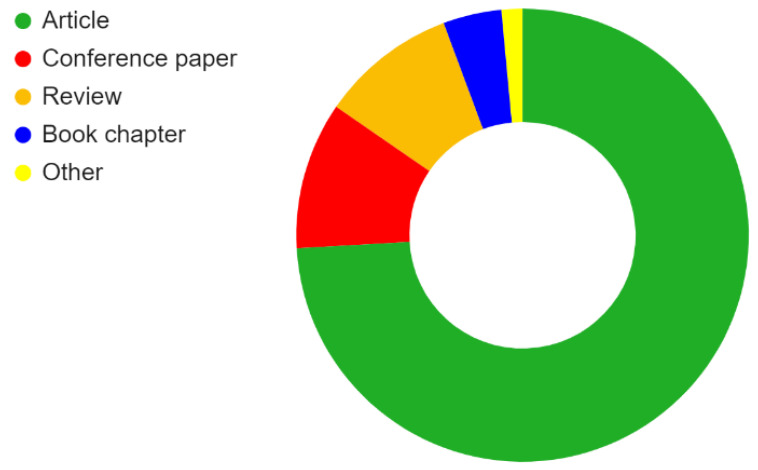
Distribution of search results from Scopus by document type.

As shown in Figure 5, articles make up about 74% of the Scopus search results. Conference papers, reviews, book chapters, and other documents account for 11%, 10%, 4%, and 1%, respectively. The large number of scientific articles in the published documents indicates that intensive research is going on in this field.

**Figure 6:**
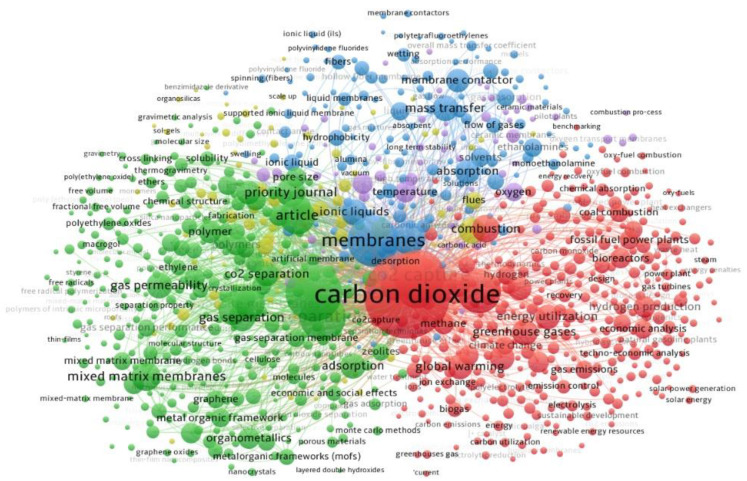
Keyword co-occurrence network visualization of search results.

The complete number of citations for scientific documents is 55,962 and the works with the largest number of citations are devoted to recent advances in CO_2_ capture and membrane separation. We imported the data into the visualizing bibliometric network tool (VOSviewer 1.6.17) to create a network map of keyword co-occurrence. A keyword co-occurrence network, also known as a semantic network, is a bibliometric data analysis method that includes a graphic visualization of possible relationships between searched keywords [29]. As can be seen in Figure 6, all keyword co-occurrences consist of 6 clusters with different colors and 978 items. 

Nodes represent keywords in the network map, whereas arcs represent co-occurrence relationships between nodes and indicate that they have become linked [30].

The most often occurring keywords, such as ‘carbon dioxide’, ‘gas permeable membranes’, ‘membranes’, ‘carbon capture’, ‘composite membranes’, and ‘mixed matrix membrane’ are shown with larger labels and nodes in each cluster.

The results of the bibliometric analysis show that scientific research in membrane separation in recent times has focused mainly on the development of membrane materials, improving membrane performances, and using membrane in hybrid separation systems.

## 3. Typical Industrial Sectors and Stream Characteristics for CO_2_ Capture

As mentioned in the introduction, energy, industry, and transportation are the largest CO_2_-emitting sectors, in which the contribution of global greenhouse gas emissions reached almost 90% in 2019 [31]. Massive electrification of transportation points has great potential to reduce the CO_2_ footprint from this sector in contrast to the additional increase in the demand for power generation [32]. As a result, energy and industrial facilities keep their domination as substantial CO_2_ emission sources that can be captured and utilized by CCU technologies.

Based on the fuel type, CO_2_ emitters in power generation involve coal, oil, and natural gas-fired power stations, and waste-to-energy power plants. Apart from these, the industrial point sources of CO_2_ constitute cement plants, crude oil refineries, iron, steel, and petrochemical factories [33]. Table 1 shows the typical industrial sectors and stream characteristics of these sectors.

Referring to CO_2_ capture from power plants and industrial sectors, the characteristics of the outlet flue gas stream are one of the main factors to be considered regarding the amount of CO_2_ in the stream. The pressure of the exhausts is nearly the same for all types of power plants at atmospheric pressure, in contrast to the different levels of temperature depending on the various conditions. For instance, in natural gas-fired combined cycle power plants, the temperature is not lower than 90 °C, in order to avoid condensation and damaging the chimney, and not above 110 °C, in order to utilize as much heat as possible. However, for most CO_2_ separation applications, the flue gas is required to be between the temperatures of 40–60 °C. In terms of stream compositions, coal- and oil-fired power stations have similar products of combustion with higher amounts of impurities such as SO_x_ and NO_x_ in comparison with natural gas combustion products, which are relatively clean with trace amounts of NO_x_. Since the CO_2_ content in the flue gas plays a vital role in the capture cost [33,34,42], coal- and oil-fired plants have advantages, with higher amounts of CO_2_ content, at an average of 12–14 mol%, compared to natural gas-fired power plants and combined cycle counterparts, at an average of 8–9 mol% and 3.5–4.5 mol%, respectively. From this perspective, the cost of CO_2_ capture for combined cycle power plants is more expensive than others due to the low CO_2_ concentration in the stream, although the efficiency of the plant is the highest. Coal-fired power stations have, however, the largest contribution to global CO_2_ emissions, at around 10 Gt annually among all other types of power plants.

As for the cement industry, since the CO_2_ emissions come from the energy-intensive limestone calcination process [58] and the combustion of fossil fuels, the cement plant flue gases have a high carbon content, at an average of 18–22 mol%. The temperature of the flue gas in cement production can be between 150–350 °C depending on the type of raw material and preheating stages. High-temperature flue gas must also be utilized with appropriate techniques to cool down it prior to flue gas decontamination. The steel production industry is the largest fuel consumer and emits a high CO_2_ content in exhaust gas, similar to cement production. Steel and cement industries are responsible for 14–19% of global greenhouse gas emissions (annually, 2.6 and 2.3 Gt CO_2_ emissions, respectively). For this reason, methods and techniques of decarbonization in these industries are being developed. When it comes to other CO_2_-emitting industries, they also emit CO_2_ resulting from fuel combustion and chemical reactions. However, it can be seen that the annual CO_2_ emissions are much lower compared to fossil fuel power plants, cement, and steel industries on a global scale.

## 4. Membrane-Based CO_2_ Capture Technologies

### 4.1. Membrane Separation

**Figure 7:**
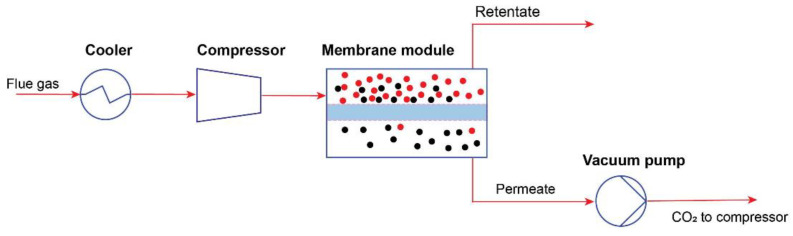
Flow diagram of basic membrane technology for CO_2_ capture (Based on [22]).

The membrane separation process is a process that uses a special module called a membrane to separate gases in a gas stream by rejecting contaminants (retentate) and passing desired components (permeate) through the membrane module shown in Figure 7. In this process, pre-treated flue gas containing CO_2_ is sent to the high-pressure side of the membrane, and CO_2_ is recovered from the low-pressure side. 

**Figure 8:**
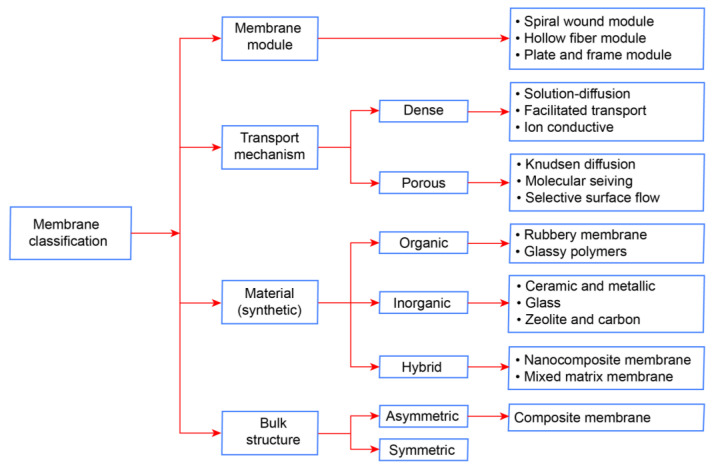
Different classifications and applications of membranes in post-combustion CO_2_ capture (Based on [67]).

Membrane separation could be a promising technology with operating parameters that go beyond current technologies, as they often feature a small footprint, easy scaling, integration into existing technologies, low operating costs, as well as low energy consumption. Membrane separation in PCC is expected to be a technology that can compete with benchmark absorption. There are mainly three types of membranes based on its material, which are organic (polymeric), inorganic (non-polymeric), and mixed matric membranes (hybrid organic and inorganic) [65]. Apart from that, membranes can also be used as a membrane contactor, enhancing the solvent-based CO_2_ capture processes. Regarding the performance of the membrane, it is highly related to the selectivity and permeability, which is the rate of passive diffusion of molecules through the membrane. In this CO_2_ capture method, mainly hollow fiber, a spiral wound, and flat sheet membrane modules are used [66]. The different classifications of membrane technology are shown in Figure 8.

Even though there are several commercial applications of membranes in different fields, the number of commercial membranes special for CO_2_/N_2_ separation is limited. For instance, Kárászová et al. [68] have reviewed various applications of commercial and emerging lab scale membranes which have been tested with flue gas. They also emphasized the existing achievements and barriers of potential membranes and evaluated their conditions of competitiveness with monoethanolamine (MEA) absorption. Apart from that, Chen et al. [69] reviewed three types of polymeric, non-polymeric, and mixed matrix membranes based on pre, oxy-fuel, and post-combustion CO_2_ separation, and concluded that more pilot plant tests should be implemented under real flue gas conditions of different fuel combustion products in post-combustion CO_2_ capture.

#### 4.1.1. Organic Membranes

In post-combustion CO_2_ separation, organic membranes are prepared by polymers such as polyacetylene, polysulfone, polycarbonates, polyetherimides, polyaniline, poly(phenylene oxide), poly(ethylene oxide), and polyvinylamine. Although the polymer-based membranes are used at low temperatures, and plasticization and swelling by water are the main issues, their relatively low cost, diversity, and easy control of processing can greatly outweigh their drawbacks. Additionally, developing the polymer and the combination of the chemical elements during the membrane preparation can be manipulated, which gives an extra advantage for this type [69,70]. In terms of transport mechanism, facilitated transport and solution-diffusion (non-facilitated transport) membranes are reported as the most widely applied and recognized in post-combustion carbon capture [65]. In the solution-diffusion transport mechanism, CO_2_ dissolves into the dense membrane followed by its diffusion throughout it. This mechanism is usually divided into rubbery, glassy, and co-polymeric membrane types which have different gas separation performances. For instance, rubbery polymeric membranes have higher permeability with inadequate selectivity, while the glassy type has opposite characteristics [71]. As for the facilitated transport membranes, CO_2_ molecules are attached by reactive carriers, forming a temporary product via reversible chemical reaction. Unlike solution-diffusion transport, facilitated transport membranes have relatively higher selectivity and permeability due to the enhancement by both aforementioned transport mechanisms [72]. Facilitated transport membranes are seen as one of the promising technologies for the flue gases from both power and industrial sectors, owing to their ability to separate CO_2_ in low partial pressure.

#### 4.1.2. Inorganic Membranes

Non-polymeric membranes are usually based on ceramic, metal, glass, carbon, and zeolite, which can practically provide better chemical and thermal stability than those of polymeric counterparts. For instance, alumina, titania, and zirconia are considered as the best choice for higher temperatures and harsh conditions, in spite of their relatively high cost [73,74]. Regarding the separation of CO_2_ from flue gas, mainly N_2_, since the dipole moments of both CO_2_ and N_2_ are zero, the ion transport mechanism is not applicable in this case. It should also be highlighted that their kinetic diameters are quite similar in size, which are 0.333 nm and 0.357 nm, respectively. Inorganic membranes can be mesoporous, microporous, and dense in type with regard to the purpose of use. On the one side, the permeability of microporous membranes is low with higher selectivity, while the characteristics of mesoporous membranes are opposite. Dense membranes, on the other side, have superior performance with their selectivity compared to mesoporous and microporous, though their permeability is lower [75]. According to Li et al. [76], several types of inorganic membranes, particularly zeolites, have reached the commercially interesting area. However, further cost reduction is needed to deploy them commercially in CO_2_ capture processes. Moreover, the processing of inorganic membranes is challenging and they tend to break easily without plastic deformation.

#### 4.1.3. Mixed Matrix Membranes

Many efforts to reach better performance of selectivity/permeability trade-off relationship on Robeson upper bounds have led to the fabrication of new a membrane technology by hybrid organic and inorganic (mixed matrix) membranes, further improving the polymeric membranes. Mixed matrix membranes are prepared from a polymer matrix filled by inorganic fillers, such as carbon nanotubes [77], metal organic frameworks [78], and zeolites [79], enabling them to take an advantage of both organic and inorganic membrane properties. Several review papers have analyzed the mixed matrix membranes and compared them to the traditional polymeric counterparts. For example, Kamble et al. [80] thoroughly reviewed and analyzed the recent works in the field of mixed matrix membranes and their inorganic fillers, emphasizing advances and the current problems of inorganic fillers materials. They also discussed the advantages and drawbacks of organic, inorganic, and mixed matrix membranes in the following Table 2.

### 4.2. Hybrid Membrane Systems

#### 4.2.1. Membrane Contactors

**Figure 9:**
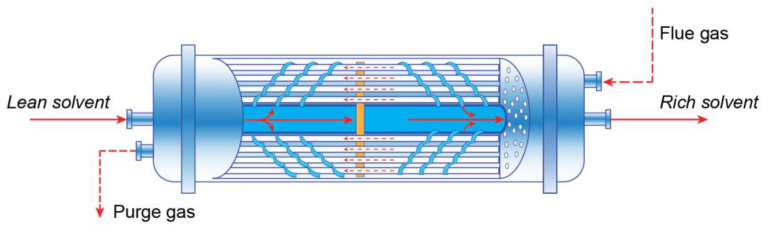
Schematic description of mass transfer in hollow fiber membrane contactors (Adapted from [90]).

Another application of membranes in CO_2_ capture processes is membrane contactors, which are a combination of membranes with solvent sorption. Membranes can be used at the gas–liquid interface, separating two phases by allowing only CO_2_ molecules pass through the membrane (dense or microporous) to the solvent side (See Figure 9) [81]. Generally, the hollow fiber and flat-sheet membrane contactors are two of the most researched technologies. In practice, the hollow fiber module is commonly used. In terms of the type of membrane material, polymeric or inorganic membranes could be chosen in response to the conditions applied. Since the process is based on the combination of membrane and solvent absorption, there are some requirements for the selection of membrane material and appropriate absorbent, including the limitations of both technologies. For instance, the selected material should provide the features of high hydrophobicity to minimize the wetting effects, thermal and chemical stability to maximize the durability, and high porosity to minimize the mass transfer resistance [82]. Absorbent characteristics also play an important role, as they have a direct influence on the process efficiency and economic aspects [83]. The commonly used solvents in this process are alkanolamines [84], amino acid salts [85], inorganic solvents [86], ammonia [87], and ionic liquids [88,89].

Membrane contactors are considered a promising technique since they allow for the avoidance of several issues such as channeling, flooding, and foaming in the conventional packed and tray columns. Apart from that, the equipment size of the column can significantly be decreased, up to 70% in size and 66% in weight, generating 4–15 times higher mass transfer area per unit volume over the traditional technique [81,91]. However, in addition to wetting and fouling of the membrane, one of the main problems of this method is extra resistance in mass transfer due to the availability of the membrane between these two phases.

#### 4.2.2. Hybrid Membrane-Absorption

Another possible application of membranes is to use them as an additional unit in the absorption process in order to improve the driving force of the mass transfer, further concentrating CO_2_ in the flue gas. This hybrid technique was initially studied by the University of Texas at Austin in collaboration with Membrane Technology and Research [92]. They integrated the selective membrane recycle unit into the absorption process in series and parallel methods. According to the results, through the best parallel configuration, the size of the absorber and flue gas flowrate can be reduced by nearly half, increasing the CO_2_ content in the flue gas from 13% to above 23%. Apart from that, several other studies have been undertaken in this field with respect to design and operational variations [93,94], economic cost evaluation [95], and possible applications in natural gas combined cycle (NGCC) power plants in selective exhaust gas recirculation (SEGR) [96,97]. Overall, this technique can be a feasible option for low CO_2_ partial pressure flue gases, particularly coal-fired and NGCC power plants (around 4% and 12–14% by volume, respectively), as long as more pilot plant tests are implemented under the real flue gas conditions.

## 5. Comparison of Membrane and Other Technologies for Post-Combustion CO_2_ Capture 

This section compares the opportunities and challenges, technological maturity, and scalability of post-combustion membrane CO_2_ separation with other alternatives. This is the best assessment to determine the current status of membrane separation technology for post-combustion CO_2_ capture by comparing it with other available methods. Table 3 summarizes the overall advantages and disadvantages of different post-combustion CO_2_ capture technologies.

### 5.1. Technology Readiness Level and Scalability

**Figure 10:**
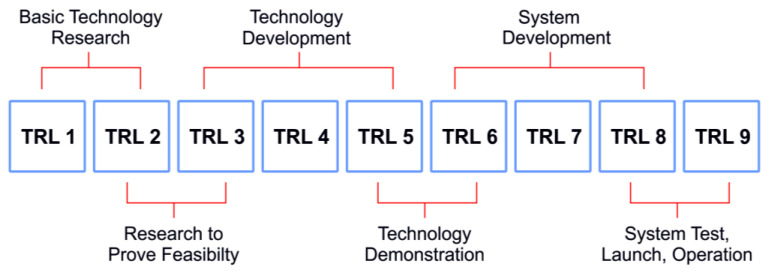
General schematics of TRL assessment (Based on [101]).

Technology readiness levels (TRL) are the technological maturity assessment of the technology developed by The National Aeronautics and Space Administration (NASA) that dates back to its origin in the 1970s. TRLs are divided into nine levels, starting from initial observations and concepts at TRL 1 and at TRL 9, at which the technology is the most mature [101]. The general schematic of the TRL assessment is shown in Figure 10. The TRL levels of various post-combustion CO_2_ capture technologies are summarized in Table 4. In addition, the current and near-future scalability potentials of these technologies are evaluated based on recent information.

Although carbon capture and storage technology (CCS) is one of the solutions to the climate change problem, there are several challenges from an economic point of view involving the interconnection between the emitting points and CCS technology. According to Global CCS Institute [102], there are 30 commercialized carbon capture, storage, and utilization facilities with a global CO_2_ removal potential of around 43 million metric tons of CO_2_/year. In addition to this, 164 other CCS projects are in the stage of construction and development. If all announced CCS facilities are launched, the potential of CO_2_ removal will increase multiple times. However, several factors, such as the current global economic crisis, the relatively low cost of CO_2_, project installation, and operating costs, may lead to the suspension of the efforts to launch these facilities. For example, the Petra Nova CCS project, capable of 1.4 million metric tons of CO_2_ removal annually, was shut down on 1 May 2020 due to the significant decrease in the price of oil, although around 1 billion USD was already spent on this project [103]. 

**Table 4:** Scalability and TRL performances of CO_2_ capture technologies.

Method	Scalability	Comment	TRL	Reference
Absorption				
Amine-based absorption	Large	Amine-based absorption (monoethanolamine (MEA), methyldiethanolamine MDEA) was fully commercialized and used on large scale (power and industrial sector)	9	[104]
Chilled ammonia process (CAP)	Large	The chilled ammonia process was demonstrated using flue gas streams (16% and 3.6% CO_2_)	7	[105]
Ionic liquid absorption	Early to assess	Although there are a few field trial tests, research and developments are mainly at lab scale	2–4	[106,107]
Piperazine solvent	Large	Piperazine (PZ) solvent for capturing CO_2_ from 4% CO_2_ flue gas was tested at natural gas combined cycle (NGCC) power plant	7–8	[108]
Phase-change solvent absorption	Small & medium	Phase-change solvent absorption was tested at packed bed pilot plant	4–5	[109]
Adsorption				
Vacuum swing adsorption/Pressure swing adsorption	Large	Although this adsorption method has already been commercialized by Air Products in hydrogen production plant, in post-combustion CO_2_ capture, it is not mature yet	2–5	[110]
Temperature swing adsorption	Medium	Large pilot tests to FEED studies for commercial plants	5–7	[111,112]
Electric swing adsorption	Early to assess	This technique is still at lab scale	3	[113]
Membrane separation				
Polymeric membranes	Small & medium	Polymeric membranes for post-combustion CO_2_ separation are in transition from pilot scale to demonstration with high possibility of commercialization	6–7	[114,115,116]
Inorganic membranes	Early to assess	Due to the complexity of material processing and relatively high cost, this membrane is mainly tested at lab-scale	3	[117]
Membrane contactors	Medium to large scale	Most of the promising membrane contactors at lab scale development with a few of them has been tested at pilot scale	4–6	[108,118]
Hybrid membrane-absorption	Medium to large scale	Hybrid membrane-absorption is being evaluated to achieve low-cost CO_2_ capture than traditional amine-based capture system. Especially for low CO_2_ concentration flue gas	3–4	[106,119]
Polymer mixed facilitated transport membranes	Small to medium scale	The pre-pilot field testing was implemented at the cement industry	6–7	[120,121]
Other post-combustion carbon capture technologies				
Cryogenic packed bed capture/Anti-sublimation system	Small to medium scale	Although this technology is mature for CO_2_/CH_4_, it is still uncertain to apply for post-combustion flue gas	3–4	[122]
Calcium looping	Large scale	Demonstrated pilot plant using oxy-fuel calcination	5–7	[123,124]
Carbon bio-fixation	Medium to large scale	Microalgae cultivation and biomass co-firing for power generation	4–6	[34,125]

TRL levels are summarized focusing on only post-combustion carbon capture performances. Current and near future scalability potential is assessed for each technology with respect to the evaluation of references and individual estimation.

In this section, since the cost estimation of CCS technology relies on industry and fuel type, flue gas contents, retrofitting opportunities, carbon capture method [126], utilization and storage pathways [127], and many other factors, we will mainly discuss the TRL level and scalability performance of post-combustion carbon capture and utilization and their comparison by each technology in the subsequent sections. 

Post-combustion carbon capture based on an amine absorption technology is fully commercialized (TRL 9) for large-scale applications and is used as a benchmark rather than other liquid solvents, piperazine (PZ), chilled ammonia, ionic liquids, alkaline solutions, and blended solvents [128], and other capture technologies. Amine, mostly MEA, absorption needs a significant quantity of heat for rich solvent recovery and power for CO_2_ compression, as well as for electrical equipment. The CO_2_ capture cost for the absorption process ranges between 50 USD CO_2_/ton and 100 USD CO_2_/ton depending on industry and solvent type [129,130,131]. The second-generation post-combustion absorption technologies involve the PZ solvent-based absorption (TRL 7-8) and the chilled ammonia processes (CAP) (TRL 7). The PZ chemical absorption has been tested at the NGCC power plant and is ready to capture CO_2_ in a large-scale application. The CAP is moving toward the commercialization stage after testing in different flue gas streams and is suitable for large-scale applications. An ionic liquid (IL) absorption technology is still in the research and development stage (TRL 2-3). ILs should be developed to overcome challenges such as toxicity, solvent cost, viscosity, low absorption capacity, corrosive nature, and hygroscopicity. Novel phase-change solvents (TRL 5-6) are currently being developed at a low-rate pilot scale in a relevant environment [109] and are expected to be available for commercialization in the next years.

While cryogenic separation for post-combustion carbon capture is the best technology to obtain pure CO_2_ in liquid or solid form, it is not yet scaling up (TRL 3-4) in post-combustion carbon capture due to the high demand for energy in the low CO_2_ composition [108,122]. Apart from that, flue gas impurities, particularly water, need to be removed in order to avoid blockage issues caused by solid ice formation of water at low temperatures, which further increases the cost of separation. Therefore, it should be noted that this technology might only be feasible when the cold energy source, such as liquefied natural gas vaporization process, is available at near locations [117].

The calcium looping process can capture CO_2_ from a large-scale power plant and other industrial flue gases [132]. Due to the high temperature of the processes in the carbonator/calciner fluidized bed reactors and additional requirements for oxygen, there are difficulties in implementing the calcium looping process from an economic point of view. For instance, in carbon capture from natural gas combined cycle power plant flue gases, the CO_2_ capture cost of calcium looping is between 90 USD CO_2_/ton and 100 USD CO_2_/ton, which is significantly more than the benchmark amine (MEA) capture process [133]. However, this technology seems more attractive because of the inexpensive natural limestone, the possibility of diverting used CaO to cement production, power/steam generation from waste heat, and its being much less hazardous to the environment compared to solvents. 

Regarding CO_2_ bio-fixation, CO_2_ can play a crucial role in boosting algae and crop cultivation. Microalgae photosynthesis, from the scalability context, is possibly assessed as a medium or even higher scale technology, considering its significant limitations including large space requirement, wastewater availability, algae sensitivity to the impurities, and high cost of control. Unavailability of sunlight at nighttime also affects the efficiency of CO_2_ removal. However, it is considered as the best-fitting technique for flue gas streams with relatively low CO_2_ content, such as the flue gas from NGCC power plants, without affecting the efficiency of the plant [134]. CO_2_ consumption in greenhouses is becoming another trend for yield boosting in many countries. For instance, the Netherlands stands out as a country in which CO_2_ is used in greenhouses up to 6.3 Mt per year [135]. Although carbon bio-fixation is generally at low TRL levels [100,125], its estimation for the end of this decade is relatively large, as the demand for biofuels and bio-based feed products rises [136]. 

The adsorption process is assessed as a viable method for gas purification. When it is implemented in PCC, challenges occur related to flue gas characteristics. In terms of pressure-based sorbent processes, the suitability of these processes to CCS highly depends on CO_2_ content from an economic point of view. An energy penalty will be significantly higher at low CO_2_ partial pressure sources than other high CO_2_ partial pressure gas streams (hydrogen production, steel, and other industrial sectors) for PSA/VSA adsorption. Although the CCS facility based on a VSA (VPSA) was demonstrated (TRL 8–9) by Air Products at the steam-methane reformers (CO_2_ from syngas), this technology can be a feasible option to commercialize for post-combustion CO_2_ capture with higher CO_2_ concentration flue gases [108]. The temperature swing adsorption (TSA), particularly the Kawasaki Carbon Capture System [112] and Svante VeloxoTherm^TM^ Rapid Cycle Temperature Swing Adsorption [106], reached a demonstration-scale with innovative sorbents and adsorption reactors. In other emerging CO_2_ capture technologies by adsorption (TRL < 5), scientific research and development are being conducted in order to solve problems depending on the limited scale of sorbent capacity, selectivity under realistic pressure conditions, moisture sensitivity, and slow kinetics. In terms of adsorbent materials, zeolites and carbon-based solid sorbents are more mature than other adsorbents and are widely used in large-scale applications [137].

Membrane gas separation is becoming one of the promising options in CO_2_ capture from fuel combustion flue gases. Although polymeric membranes have already been commercialized in natural gas processing [106], in post-combustion CO_2_ separation, there are only three polymeric membranes so far that have been demonstrated on a pilot scale reaching the level of TRL 5–6. These are Polyactive^®^ membrane by Helmholtz-Zentrum Geesthacht in Germany, Polaris^®^ membrane by Membrane Technology and Research Institute in the USA, and fixed-site-carrier membrane by the Norwegian University of Science and Technology in Norway [116]. Nevertheless, there are still some major issues remaining when tested under real flue gas conditions. These problems include humidity-based membrane resistance, thermal influences on transport properties (permeability and selectivity), stability of the membrane layer for a long period, and the impurities of the flue gas [68]. However, in the last decade, significant progress has been made in CO_2_ capture using polymer matrix membrane processes. According to Janakiram et al. [115,121,138], water content in the flue gas is no longer an impurity, but it is a promoter. Humidity in the module can increase membrane permeability. They tested hollow fiber modules of hybrid facilitated transport membranes for flue gas coming from the cement industry. Membrane performance improved when there was more water in the flue gas stream. As for the inorganic membranes, they are still in the lab scale development, with TRL 3–4 [117], due to several problems related to the permeability of dense membranes, difficult processing, and high cost, as mentioned in the previous section. According to Jusoh et al. [139], the fabrication cost of membrane modules for zeolite is 150 times higher than polymeric hollow fiber membrane modules, at 3000 USD and only 20 USD per square meter, which leads to its limitations in industrial applications. In terms of hybrid membrane-absorption techniques, on the one hand, there are several field trial and pilot scale studies of membrane contactors in CO_2_ capture. However, membrane wetting and its compatibility with solvent still remain as the major issues. For instance, the latest pilot study of membrane contactors, reported by Scholes et al. [118], revealed that membrane contactors can be a feasible option for industrial applications, though the energy consumption in the pilot study is higher than conventional capture technique (less than 4.2 MJ/kgCO_2_) due to thermal losses in membrane modules and energy integration issues. Membrane separation integrated absorption technology, on the other hand, was assessed at a TRL of 4 as a conceptual study by the Global CCS Institute in 2021 [106]. Freeman et al. [119] conducted a bench scale study of hybrid membrane-absorption CO_2_ capture from coal-fired flue gas. In accordance with this report, in the hybrid system, CO_2_ concentration in the flue gas can be increased to around 20% using an MTR air-swept Polaris™ membrane module with 15% lower capture cost than the conventional amine technology result of the National Energy Technology Laboratory.

### 5.2. Overall Technology Comparison for CO_2_ Capture

Here is a general discussion about five different CO_2_ capture technologies and their comparison based on CO_2_ purity and recovery, scalability, TRL, capital expenditures (CAPEX), operational expenditures (OPEX), and environmental benefit without certain metrics. In order to carry out the best realistic comparison among these technologies, there are many factors and conditions to consider for each specific case and technology that makes the work more challenging. Therefore, the following discussion and comparison are conducted to make a general overview of the key highlights of each technology. 

Regarding the captured CO_2_ recovery and purity, the cryogenic method is evaluated as the most suitable technology, capable of 99.99% product purity, since this process is based on obtaining CO_2_ in liquid or solid form at a very cold temperature. This technology has been applied in mainly air separation units, blue hydrogen production, natural gas processing plants, and biogas processing so far. Nevertheless, the challenges that the process is energy intensive and requires a high concentration of CO_2_ in the flue gas stream are hindering this technology’s commercial deployment and scalability assessment in the post-combustion field. Absorption, calcium looping, and adsorption (TSA) also have great potential to obtain a relatively pure CO_2_ product, but the equipment cost and operating cost also increase. Membrane separation and adsorption (VPSA) processes are not favorable in this context due to the necessity for multiple stage installation, which leads to additional CAPEX and OPEX. CO_2_ bio-fixation can be assessed for only CO_2_ recovery rate, which is quite a bit lower than other techniques, since the process directly utilizes the captured CO_2_.

From the scalability point of view, absorption by amines can be evaluated as the most reliable technology and capable of capturing CO_2_ at a large scale, followed by the calcium looping process, which is not fully mature yet. For example, Zanco et al. [140] performed a comparative study of absorption, adsorption, and membrane technologies, selecting the most mature and effective techniques under the same condition for all. According to the results, adsorption and membrane technologies are more cost competitive than the absorption process in terms of small-scale plants. However, at large scale plants and higher CO_2_ recovery rates, absorption is found to be the most cost-effective. Carbon bio-fixation can also be applied for large scale power plants without a need for flue gas pre-processing.

**Figure 11:**
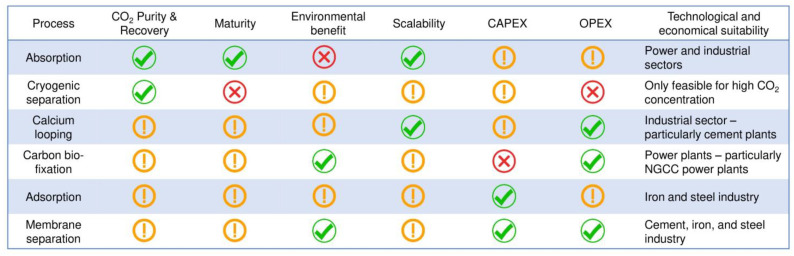
Post-combustion CO_2_ capture methods comparison based on five different parameters. This individual comparative assessment is just to identify the relative key highlights of different PCC methods and is not intended to make a final decision. Tick in green, exclamation in yellow, and cross in red marks represent good, neutral, and bad, respectively.

When the TRL levels are considered, as discussed previously, the number of techniques that have reached maturity is higher in the absorption process than others, followed by adsorption techniques. However, membrane separation (polymeric and mixed) is assessed as the fastest developing technology due to its wide range of characteristics that have not yet been studied well compared to the cryogenic separation method, which attracts the least attention in terms of commercial deployment in PCC. The following is Figure 11, which provides an overview of different technologies’ comparison based on five categories. 

Regarding the overall CAPEX and OPEX, facilitated transport membranes (polymer mixed) can be more potent than other techniques due to their acceptance of water, which makes them unique among other membrane types [138], although this membrane is not commercially available and not fully mature yet. As membrane science has been developing rapidly in recent years, its advantages, such as easy scale-up, small footprint, and, particularly, lower energy consumption, can overcome the conventional benchmark absorption technology. In terms of carbon bio-fixation, this technique requires very high capital investment due to the large number of photobioreactors’ installation and large area requirements. In contrast, OPEX is relatively low, as the process goes at ambient conditions. Ca-looping also seems acceptable from the OPEX, since the sorbent (CaO) is relatively cheap, but the additional air separation unit leads to an increase in the CAPEX. However, in the case of amine scrubbing and solid sorption, the CAPEX and OPEX highly depend on which type of solvent or sorbent and techniques are used, their scalability, availability at low cost, site conditions, maturity level, and many other factors. For instance, in the MEA absorption process, the majority of the OPEX is connected to the solvent regeneration energy and its heat integration to the point source, followed by solvent loss and its degradation, while CAPEX varies in response mainly to the dimensions of the columns, packing or tray type, and heat exchangers based on the flowrate, CO_2_ partial pressure in the flue gas, purity requirement of CO_2_, and capture rate.

As for the environmental concerns, it is very difficult to evaluate without a rigorous lifecycle analysis of each technology. However, in general, carbon footprint of absorption with amines can be higher than other techniques since there is an extra emission of solvent, which has an even higher impact on the environment than CO_2_. Solid sorption, calcium looping, and cryogenic separations also possibly have more environmental stress (depending on the energy intensity and its source) than membrane separation and carbon bio-fixation, which are both considered as the most energy-efficient, with less of a carbon footprint.

## 6. Conclusions and Future Works

In this study, the recent research on membrane-based post-combustion capture and other PCC technologies has been discussed in terms of the types of technology available and their potential contribution to the abatement of CO_2_ emissions. In addition, the existing challenges and opportunities of using membranes in the decarbonization of power and industrial sectors are discussed. 

In recent decades, CO_2_ emissions have reached their highest level, with an increase of nearly 50% related to human activities. Therefore, CCSU plays a crucial role in line with renewable energy to reduce CO_2_ emissions. 

Regarding the results of the bibliometric analysis, scientific research in the field of membrane-based CO_2_ separation in recent years has focused mainly on the development of membrane materials, improving membrane performances, and using membranes in hybrid separation systems.

Aside from this, several important generalized conclusions can be drawn from this review:

- Stationary CO_2_ emitters from the power sector and CO_2_ sources from industry are the first places where their emissions should be reduced.
- CO_2_ content in flue gas plays a vital role from a technological and economical point of view.
- According to current studies, membrane technology for PCC will play an important role in the near future, since there is a high possibility for further investigations of new membrane materials and optimization of existing ones.
- The study comparison shows the overall best result for membrane technology in different categories, such as CO_2_ purity and recovery, maturity, scalability, CAPEX, OPEX, and environmental benefits.
- As membrane studies on decarbonization of large emitters have been developing rapidly in recent years, its advantages involving small carbon footprint, easy scale-up, and, especially, lower energy consumption are expected to overcome the first-generation benchmark absorption technology.
- Despite the scientific progress achieved, there are still challenges in the implementation of membranes in real conditions for PCC. These issues include humidity-based membrane resistance, thermal influences on permeability and selectivity, long-term stability of the membrane layer, and the tolerance for flue gas impurities.
- The membrane gas separation method can be suitable for flue gases with higher CO_2_ content, such as cement, iron, and steel industry.
- Large scale application of membrane technology, particularly in power plants, can be challenging due to the low partial pressure of CO_2_ in the flue gas. To overcome this issue, it is essential to develop a membrane material with high selectivity and permeability, ease of fabrication, and low cost.
- In recent years, several advances have been made and tested in real conditions regarding the effect of flue gas impurities on membranes. However, there is still a need for further research to find a membrane material with optimal characteristics for PCC.

Based on this review, we can conclude that the current low price of the CO_2_ market and the relatively high cost of CO_2_ separation (particularly regeneration energy) prevent PCC techniques from deployment to large-scale point sources. In this case, today’s global efforts to reduce the emissions by CCSU have turned to improving the highly performed existing membrane materials and investigating novel, low cost membrane separation techniques, along with CO_2_ valorization.

## Figures and Tables

**Table 1:** Typical industrial sectors and stream characteristics. (modified from [34]).

Power Generation Sector	CO_2_ Content	Global CO_2_ Emissions (Mt/Year)	Capture Cost ($/tCO_2_)	Flue Gas Temp (°C)	Flue Gas Component	Reference
Power sector						
Coal-fired power plant	12–16	9900	41–100	40–80	CO_2_, CO, O_2_, N_2_, H_2_O, NO_x_, SO_x_	[35,36,37,38,39,40,41,42]
Natural gas-fired power plant	7–10	6336	41–100	90–178	CO_2_, O_2_, N_2_, H_2_O, trace amount of NO_x_	[42,43,44]
Power plant with natural gas combined cycle	3–6.5	6336	50–100	90–110	CO_2_, O_2_, N_2_, H_2_O, trace amount of NO_x_	[42,45,46,47,48]
Oil-fired power plant	12–14	755	58–100	N/A	CO_2_, O_2_, N_2_, H_2_O, NO_x_, SO_x_	[32,42,49]
Cement, iron and steel industries						
Cement production	14–33	2310	60–120	150–350	CO_2_, CO, N_2_, H_2_O, NO_x_, SO_x_	[42,46,50,51,52,53,54,55,56,57,58]
Iron and steel industry	15–27	2632	40–100	~100	H_2_, CO_2_, CO, N_2_, H_2_O, H_2_S	[42,59,60]
Other industrial sectors						
Refineries	3–20	>1000	35–100	160–190	Depends on the fuel used (commonly CO_2_, CO, O_2_, N_2_, H_2_O, NO_x_, SO_x_, Ar)	[42]
Ammonia production	18	450	25–35	N/A	H_2_, CO_2_, CH_4_, O_2_	[32,42,61,62]
Hydrogen production	15–20	830	50–80	N/A	H_2_, CO_2_, CH_4_, CO	[32,42,63]
Methanol production	10	222	40–60	~141	Mainly CO_2_, O_2_, N_2_, H_2_O	[32,49,64]

**Table 2:** Comparison of different characteristics of organic, inorganic, and mixed matrix membranes [80].

Properties	Organic Membranes	Inorganic Membranes	Mixed Matrix Membranes
Fabrication cost	Low	High	Moderate
Chemical and thermal stability	Moderate	High	High
Synthesis and processability	Easy	Difficult	Easy
Plasticization	Susceptible	Insusceptible	Insusceptible
Surface roughness	Low	High	Moderate
Fouling resistance	Low	Moderate	Moderate
Cleaning after fouling	Difficult	Easy	Easy
Swelling	Frequently occurs	Swelling-free	Swelling-free
Resistant to pressure	Moderate	High	High
Mechanical strength	Good	Poor	Excellent
Gas Separation performance	Below the Robeson’s upper bound	Above the Robeson’s upper bound	Above the Robeson’s upper bound

**Table 3:** Overall advantages and disadvantages of CO_2_ capture technologies.

Advantages	Disadvantages
Membrane separation	
High separation efficiency can be achieved Fast development possible due to modular set-up Promising technology for low energy consumption and lower capital cost compared to conventional separation technologies Easy scale-up Smaller footprint Easy for remote area	Swelling of the membrane by water O_2_ and sulfur dioxide (SO_2_) might pass through the membrane with CO_2_ High energy for compressions Limited purity of CO_2_ Lowering membrane area demand is challenging
Hybrid absorption-membrane separation	
High CO_2_ concentration of flue gas can be achieved Lower capital cost due to higher concentration Energy consumption can be minimized	High operational/maintenance cost Flue gas pre-treatment considering membrane and absorbent characteristics
Amine scrubbing (absorption)	
Most mature technology Capturing level is high enough (80–95%) Solvent can be regenerated above 95% Applicable to large scale CO_2_ product purity above 98% Applicable to flue gases with low CO_2_ partial pressure	Solvent degradation Regeneration requires a high amount of energy Corrosive environment Emissions by degradation of the solvent
CO_2_ bio-fixation	
No particular feed stream quality is required A source of energy capable of replacing fossil fuels (biofuels) Efficient in low CO_2_ concentration Captured CO_2_ can directly be utilized as bio-product Wastewater can be used Environmental benefit	Algae sensitive to impurities, pH Expensive to control growing and drying processes Large space is required Water resource necessity Saturation of microalgae to CO_2_ takes long time Only feasible to partial CO_2_ removal Sunlight unavailability at nighttime
Adsorption	
Adsorbents can be recycled High capture efficiency Resistant to long-term use Lower environmental impact VPSA (vacuum pressure swing adsorption) can be highly energy-efficient at higher CO_2_ partial pressure	Flue gas pre-treatment (cooling and drying) Desorption process is energy-intensive Negative impact of SO_x_ and NO_x_ on adsorbents No ideal adsorbent Maturity is low in PCC
Cryogenic separation	
Highly purified products There is no need for additives or chemical reagents CO_2_ can be obtained in liquid form ready for transport High CO_2_ recovery rate can be achieved	Refrigeration requires a high amount of energy Only viable for high CO_2_ concentration above 90% Flue gas should be dehydrated
Calcium looping	
Energy efficiency loss is low (4–7%) Highly developing technology for cement industry owing to waste heat recovery	Attrition depending on raw meal/limestone hardness Calcination process requires additional fuel burning Air separation unit is required to fuel-burning of calcination Reactivity efficiency of CaO decreases in multiple carbonation/calcination cycles
[33,34,72,98,99,100]	

## Data Availability

Data sharing is not applicable.

## Abbreviations

Ar	Argon
C	Carbon
CaCO_3_	Calcium carbonate
CaL	Calcium looping
CaO	Calcium oxide
CAP	Chilled ammonia process
CAPEX	Capital expenditures (capital cost)
CCS	Carbon Capture, and Storage
CCSU	Carbon Capture, Storage, and Utilization
CCU	Carbon Capture and Utilization
CH_4_	Methane
CO	Carbon monoxide
CO_2_	Carbon dioxide
FEED	Front End Engineering Design
H_2_	Hydrogen
H_2_O	Water
H_2_S	Hydrogen sulfide
IEA	International energy agency
ILs	Ionic liquid
MDEA	Methyldiethanolamine
MEA	Monoethanolamine
N/A	Not applicable
N_2_	Nitrogen
N_2_O	Nitrous oxide
NGCC	Natural gas combined cycle
NO_x_	Nitrogen oxide
O_2_	Oxygen
OPEX	Operational expenditure (operating cost)
PCC	Post-combustion capture
PSA	Pressure swing adsorption
PZ	Piperazine
SEGR	Selective exhaust gas recirculation
SO_2_	Sulphur dioxide
SO_x_	Sulphur oxide
TRL	Technology Readiness Level
TSA	Temperature swing adsorption
VPSA	Vacuum pressure swing adsorption
VSA	Vacuum swing adsorption
